# Chikungunya, Dengue, and Zika in Immunocompromised Hosts

**DOI:** 10.1007/s11908-018-0612-2

**Published:** 2018-03-17

**Authors:** Luiz Guilherme Darrigo, Alexandre Machado de Sant’Anna Carvalho, Clarisse Martins Machado

**Affiliations:** 1 Bone Marrow Transplant Unit - Ribeirão Preto Medical School, University of São Paulo, Ribeirão Preto, São Paulo Brazil; 2 Virology Laboratory – Institute of Tropical Medicine, University of São Paulo, Av. Dr. Enéas de Carvalho Aguiar, 470 - 2nd floor, São Paulo, SP 05403-000 Brazil; 3 HSCT Program, Amaral Carvalho Foundation, Jahu, São Paulo Brazil

**Keywords:** Chikungunya, Zika, Dengue, Transplantation, Immunocompromised hosts, Arboviruses

## Abstract

**Purpose of Review:**

Describe the characteristics of chikungunya, dengue, and Zika in transplant recipients and immunocompromised hosts.

**Recent Findings:**

Stem cell/bone marrow grafts, organs, and blood transfusions can transmit CHIKV/DENV/ZIKV infections, which are clinically similar, resembling influenza-like illness. Laboratory confirmation is necessary. In the acute phase, RT-PCR is preferred. DENV and ZIKV serology may cross-react. Delayed engraftment and extended viruria is observed in ZIKV+/HSCT recipients, while longer viremia is observed in DENV+/HSCT patients. Arbovirus persistence in organ tissues is generally unknown. Vaccine development is in early stages for CHIKV/ZIKV. No data is available to recommend the licensed DENV vaccine in transplant recipients.

**Summary:**

In endemic areas, the assessment of epidemiological risk is mandatory. Donor deferral for 120 days in suspected or confirmed ZIKV+ has been recommended, while CHIKV+ donors should wait 30 days. No deferral is recommended for DENV+ donors. CHIKV/DENV/ZIKV tests should be included in the differential of febrile neutropenia and other transplant syndromes. Reassessment of DENV serology is urgently needed. Prospective studies are necessary to determine the impact of CHIKV/DENV/ZIKV in this special population.

## Introduction

Diseases caused by arboviruses may represent a major threat to immunocompromised hosts living in or traveling to endemic regions. Although dengue is the most common mosquito-borne disease in tropical and subtropic regions, two new arboviral diseases, chikungunya and Zika, have been recently introduced in the Americas. These three arboviruses have posed new challenges in the transplantation setting, such as lack of specific serological tests, possibility of blood and graft transmission, and restrictions in donor selection. The diseases have a similar clinical presentation in the acute phase, hindering appropriate diagnosis, case management, and sometimes prompting severe and fatal events.

In this review, relevant data on chikungunya, dengue, and Zika infections in transplant recipients and immunocompromised hosts is discussed.

## Dengue

Dengue is a mosquito-borne viral disease that occurs both as an endemic or epidemic disease. Dengue virus (DENV) is a small single-stranded RNA virus comprising four main serotypes belonging to the genus Flavivirus, family Flaviviridae, and transmitted by mosquitoes of the genus Aedes (*Aedes aegytpi* and *Aedes albopictus*) [1]. Infection by one serotype provides lifelong immunity against that serotype, but only partial protection against subsequent infections by other serovars. In immunocompetent hosts, there is good evidence that secondary infection increases the risk of more serious disease due to antibody-dependent enhancement (ADE), resulting in dengue hemorrhagic fever (DHF) or dengue shock syndrome (DSS). Patients with severe dengue have elevated circulating levels of IL-8, IL-10, TGF-β, and interferon-γ [2]. Therefore, a robust immunologic response is a prerequisite for the development of DHF or DSS.

### Epidemiology

An estimated 3.9 billion people in 128 countries are at risk of infection with DENV [3]. A recent study indicates that around 390 million dengue infections occur every year, of which 96 million manifest clinically with variable severity [4]. In the last 15 years, dengue outbreaks have been reported in most World Health Organization (WHO) regions. In 2016, more than 2.38 million cases with 1032 deaths were reported in the Americas, where Brazil alone contributed with approximately 1.5 million cases [5].

In the last 5 years, an increasing number of dengue has been described in transplant recipients due to the expansion of DENV [1]. So far, more than 180 cases have been reported [6••, 7, 8, 9••, 10, 11]. In endemic areas, this number may be much higher, since most cases are mild and present as a flu-like illness, with some manifestations resembling post-transplant syndromes.

### Dengue Transmission in Transplantation

More than 95% of the transplant recipients acquired dengue by vector transmission, as they were living or traveled to endemic areas [6••, 10, 11]. Although mosquito bites are the most frequent mode of transmission, other sources of infection are relevant in the transplant setting, such as the graft itself and blood transfusions.

Graft transmission is rare but has been well documented in two cases of hematopoietic stem cell transplantation (HSCT) [12•, 13]. The first case occurred during the 1994–1995 dengue epidemic in Puerto Rico. The patient died 11 days after HSCT and DENV4 was detected in blood, ascitic fluid, and tissue samples. The donor developed fever 2 days after marrow harvesting and DENV4 serotype was confirmed in donor samples [12•]. The other case of graft transmission occurred in a HSCT recipient whose unrelated donor had returned from Sri Lanka 3 days before donation [13]. Graft transmission has also been reported in solid organ transplant (SOT) recipients [14]. A living donor who developed symptoms 2 days after donation transmitted dengue to a liver transplant recipient in Delhi [15•]. Transfusion-transmitted (TT) dengue has also been demonstrated. Viremic donors have been detected during outbreaks and most TT/DENV+ cases are asymptomatic [16••, 17–19]. So far, routine NAT for dengue is not recommended in blood banks.

During dengue season in endemic areas, asymptomatic SOT/HSCT donors who live or have traveled to the region should be directed to report dengue symptoms appearing in the first week after donation. Donor deferral is currently not recommended.

### Clinical Manifestations

Dengue has a wide clinical spectrum varying from asymptomatic to severe clinical manifestations. In the immunocompetent population, seroprevalence studies suggest that the number of asymptomatic carriers is threefold to that of dengue fever cases.

**Fig. 1:**
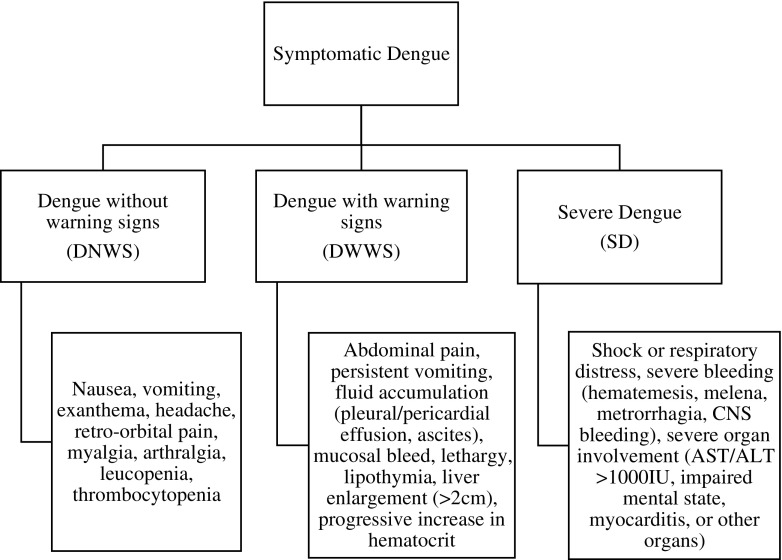
Current classification of symptomatic Dengue infection [20•]

Symptomatic dengue is currently grouped into three categories: dengue without warning signs (DNWS), dengue with warning signs (DWWS), and severe dengue (SD), as shown in Fig. 1 [20•]. The absence of warning signs does not preclude the possibility of severe disease and lethal outcome [1].

After a 3- to 15-day incubation period, the disease presents abruptly as a flu-like illness in three phases, i.e., febrile, critical, and recovery. This stereotypical course is often altered in transplant patients, where longer duration of symptoms and thrombocytopenia occur in more than 80% of cases.

A recent study including five HSCT/DENV+ patients showed main clinical findings to be fever (100%), myalgia (80%), thrombocytopenia (80%), and rash (60%), with or without hemorrhagic episodes, similar to immunocompetent individuals. Severe DHF occurred in one patient (20%). An important finding of this study was the observation of prolonged viremia (more than 15 days) in all patients who had blood samples collected serially [6••]. In the immunocompetent population, dengue viremia persists from 4 to 7 days (median 5 days) in 69% of the patients, being uncommon after the fifth day (12.5%) [21, 22].

Among SOT recipients, the largest case series was published in 2013 and reported the clinical findings of 102 DENV cases after renal transplantation [9••]. Forty-four patients (43%) had primary and 58 (56.8%) had secondary dengue infection. Thrombocytopenia was seen in 95% of cases, with a mean duration of 11 ± 9 days. Most patients presented with fever (80%), which was less frequent in patients receiving high-dose steroids. DF occurred in 88% and DHF/DSS occurred in 11.7%, with graft dysfunction in 66.7% of these. Interestingly, patients on a cyclosporine (CSA)-containing regimen had less severe disease [9••]. Some authors have shown that CSA can be a potential drug for the treatment of flavivirus infections [23•].

In immunocompetent hosts, dengue mortality rates vary from 0.026% in DF up to 5% in DHF/DSS [24]. Studies in transplant populations show mortality rates ranging from 0 to 100% in case reports and 0 to 37.5% in case series. The main publications highlighting these findings have been recently summarized [6••]. Prospective studies may better determine the morbimortality of dengue in transplant cohorts as well as the variables associated with severe forms of the disease.

### Diagnosis

After disease onset, the virus can be detected in serum, plasma, circulating blood cells, and other tissues for 4–5 days. Virus isolation, NS1 antigen detection that yields results within a few hours, or nucleic acid test (NAT) can be used to diagnose dengue in the acute phase. A recent study showed that prolonged viremia (over 15 days) is frequently observed in transplant patients, thus NAT can be used to diagnose dengue even after the first week of illness in this population [6••].

After the acute phase of infection, serology is the method of choice for diagnosis. Specific IgM antibodies are detectable in 50% of patients by days 3–5 after illness onset, increasing to 80% by day 5 and 99% by day 10. IgM levels peak about 2 weeks after the onset of symptoms and then decline to undetectable levels over 2–3 months. IgG is detectable at low titers at the end of the first weeks, increasing slowly thereafter with serum IgG still detectable after several months, probably for life [1].

In recent years, with the spread of ZIKV and CHIKV in dengue regions, cross-reactivity in serological tests has unfortunately been observed, with some Zika patients testing as false-positives for dengue [25, 26••]. As a result, both diagnosis and seroprevalence estimates are compromised.

### Clinical Management

Fever and exanthema are good markers of arbovirus infection, especially during mosquito season, and should prompt DENV, ZIKV, and CHIKV investigation [27••]. Thrombocytopenia is a hallmark of dengue and helps to differentiate it from other arboviruses [6••, 27••].

Overall assessment includes history, physical exam, laboratory tests, and evaluation of disease severity [11]. Based on the findings, clinicians are able to determine the phase of the disease, the presence of warning signs, and if the patient requires admission.

There is no specific antiviral drug to treat dengue. Generous fluid replacement is the mainstay of therapy. Seropositive patients have a risk of developing severe dengue in case of secondary DENV reinfection with different serotypes. However, this tendency has not been confirmed in transplant recipients. The T cell immunosuppression induced in this population and consequent low inflammatory response may explain these findings. Currently, there is no evidence to recommend decreasing immunosuppression in transplanted patients, as it does not seem to affect the outcome and may trigger graft rejection [9••].

## Chikungunya

Chikungunya virus is an arbovirus, belonging to the family Togaviridae genus Alphavirus. It is transmitted by Aedes mosquitoes (*Aedes albopictus* and *Ae. aegypti*) and was first isolated in the 1952 outbreak in a southern province of Tanzania [28, 29]. After limited outbreaks in Asia in the 50s and 60s, the disease re-emerged in the early 2000s and rapidly spread throughout Africa and Europe [30]. Until now, four lineages distinguished by genotypes have been identified [31].

### Epidemiology

During the 2004 epidemic, which began in Africa, several outbreaks were reported in the Indian Ocean islands. Afterwards, an outbreak occurred in La Reunion Island, affecting approximately 34% of the population. In 2006, several cases of CHIKV infection were reported in Europe [32, 33]. In 2013, cases of CHIKV infection were reported in St Martin Island and spread throughout the Caribbean, Central America, and South America [34]. In 2014, cases of CHIKV began to be identified among returning travelers from the USA who visited affected areas. Local transmission was identified in Florida, Puerto Rico, and the US Virgin Islands. In the same year, the first case of autochthonous CHIKV was identified in Brazil [35]. Between 2015 and 2017, more than 300,000 cases were notified to the Brazilian episurveillance system, with 40% of municipalities confirming cases [33, 35]. Currently, Brazil accounts for more than 90% of confirmed cases in the Americas [36].

### Chikungunya Transmission in Transplantation

Besides vector transmission, few articles discuss CHIKV transmitted by organ transplantation or blood transfusion. In the 2005 outbreak in La Réunion, CHIKV was detected in 4 of 12 healthy donors of corneal grafts, demonstrating the potential risk of transmission during tissue transplantation [37•]. Previous reports of CHIKV transmission by blood donation estimated that the mean and maximal risks of viremic donations were 38 to 52 per 100,000 donations (0.04 to 0.06%), respectively [38].

The recommendations concerning donor screening for CHIKV are controversial. Couderc and colleagues observed silent eye infection in 33% of corneal donors during the outbreak in La Reunion and demonstrated that systemic infection followed cornea infection in animal model. The authors recommend that in the absence of systematic CHIKV screening in donors, cornea donation should be banned during CHIKV outbreaks [37•]. There are no studies investigating long-term persistence of the virus in tissues, and the cost-effectiveness and clinical impact of such guidelines is subject to debate.

As the maximum viremic period of CHIKV infection is 2 days before and 17 days after symptoms [38], we suggest donor deferral for at least 30 days if SOT/HSCT donor lives or has traveled to endemic areas and presents with CHIKV symptoms or laboratory confirmation. We also recommend discussing the risk of donor-derived infection, especially in epidemic settings, and obtaining informed consent. Asymptomatic SOT/HSCT donors should be directed to report CHIKV symptoms appearing in the first week after donation.

### Clinical Manifestations

After a mosquito bite, CHIKV reaches the bloodstream and spreads through the liver, muscles, joints, spleen, lymph nodes, and brain. In immunocompetent patients, the incubation period varies between 2 and 10 days [33]. After this, an abrupt onset of fever is observed, followed by malaise, maculopapular rash, myalgia, nausea, and headache. Persistent or recurring arthralgia is characteristic and helps to differentiate CHIKV from other arboviruses [29, 39••]. A considerable number of patients develop chronic polyarthritis that persists for months or years after the acute phase. It is estimated that after 1 year, more than 20% of patients still have incapacitating joint pain [40]. Despite being a benign and self-limited disease, there are reports of neurologic and hepatic manifestations with fatal outcomes [41]. The fatality ratio is about 1 per 1000, with most cases occurring among newborns and elderly [29].

Unlike ZIKV and DENV, CHIKV is symptomatic in more than 95% of cases [29]. However, in SOT recipients, the intensity of joint symptoms may not be as expressive as in immunocompetent individuals. Kee and colleagues reported two cases of CHIKV in a chronic kidney disease patient and liver transplant recipient associated with peritonitis, encephalitis, and secondary bacterial infections, but without arthralgia, which is characteristic of CHIKV infection [42].

In SOT, Chikungunya has been reported as a mild disease with favorable outcomes [43, 44]. The largest study of SOT/CHIKV+ patients was performed in Ceará, an endemic area in the north of Brazil, where authors described 13 cases of CHIKV infection (9 kidney and 4 liver recipients) between January and December 2016. All patients had arthralgia and 84.6% had fever [45••]. All individuals achieved full recovery without complications [43–44, 45••, 46••]. Similarly, HSCT/CHIKV+ patients have been reported without increased morbidity [27••]. The use of immunosuppressive drugs may play a role in the paucity of symptoms.

### Diagnosis

CHIKV infection is diagnosed on the basis of clinical, epidemiological, and laboratory criteria. Lymphopenia is a key finding, although thrombocytopenia, increased AST/ALT and hypocalcemia may also be present [30]. The laboratory diagnosis is based on NAT in blood samples by reverse-transcriptase PCR (RT-PCR), viral culture, or detection of IgM and IgG antibodies through serological tests. Viral isolation from blood cells is the gold standard but is rarely performed due to technical difficulty [33, 39, 46••]. In the first week of symptoms, RT-PCR is the method of choice. In transplant recipients, CHIKV viremia seems to be shorter than in DENV infection [27••].

### Clinical Management

Treatment of acute CHIKV is mainly supportive [33]. Antiviral agents and monoclonal antibodies are in initial stages of testing. Wang and colleagues describe potential active compounds for treatment of CHIKV, such as Niclosamide and Nitazoxanide. Another compound, Suramin, has been shown to have anti-CHIKV action. Although promising, these results need to be confirmed in clinical trials [47]. An excellent review of potential therapeutic compounds was recently published [39••].

## Zika

Zika virus is a Flavivirus, in the family Flaviviridae [48••]. In urban environments, two species are considered the main competent vectors, *Aedes aegypti* and less commonly, *A. albopictus*, which has a wider distribution in North America [48••]. ZIKV was first identified in 1947 in a sentinel rhesus monkey in the Zika Forest of Uganda. The first cases of human infection were published in Eastern Nigeria in 1952 [49]. Since then, few cases had been published until 2007 when an outbreak occurred in the island of Yap, Micronesia, with approximately 5000 cases reported, followed by outbreaks in the French Polynesia and Gabon [50, 51]. The virus was then confirmed in Brazil in May 2015 and in a few months more than 1.5 million cases were reported [52, 53]. Following this rapid expansion through the Americas and the discovery of devastating effects of ZIKV during pregnancy, the World Health Organization (WHO) classified the epidemic as a public health emergency of international concern [54].

### Epidemiology

From May 2015 to December 2016, as much as 707,133 cases of ZIKV in the Americas were reported, with the majority coming from South America, particularly Brazil [55]. Epidemiologists believe that the introduction of ZIKV in the country was facilitated by several international sports events hosted by Brazil, such as the Confederations Cup in 2013, the 2014 FIFA World Cup, among others [56, 57]. In their wake, reports of ZIKV were documented in the USA and Europe. At the end of 2015, a ZIKV outbreak in Cape Verde marked the return of the virus to Africa [58]. Recent data from WHO shows that around 75 countries have reported mosquito-borne ZIKV transmission [54].

### Zika Transmission in Transplantation

The majority of ZIKV cases in transplant or immunocompromised patients were vector-borne. In SOT recipients, Nogueira and colleagues reported a series of four recipients (liver, 2; kidney, 2) who developed symptomatic ZIKV infection diagnosed by RT-PCR. All recovered without sequela [59••]. A case of ZIKV meningoencephalitis in a heart transplant recipient has also been reported [60•]. A recent prospective study in HSCT/ZIKV+ patients in Brazil described clinical findings and outcomes on four cases during the ZIKV epidemic in Brazil [27••].

Alternative ways of transmission include sexual, perinatal, and via blood products. Transmission by transplantation is another possibility. Simkins and colleagues in Florida observed that 3% of the deceased donors had positive ZIKV IgG, and none had a positive ZIKV IGM or PCR [61]. During the French Polynesian ZIKV outbreak, Musso and colleagues reported incidence of 2.8% of blood donors ZIKV+ by PCR at time of donation [62••]. Puerto Rico and Brazil had 0.5 to 1% of donors found to be ZIKV RNA-positive [63, 64]. The first case of Zika in a liver transplant recipient was reported in Brazil and was transmitted by the platelet component transfused during surgery. The blood donor was asymptomatic at the time of donation. Data has shown that 80% of infections are asymptomatic [62••, 65•]. Recently, transmission by platelet transfusion has been documented in two other patients [66].

Regarding deferral, US guidelines recommend that organ procurement organizations should focus on epidemiological risk factors, as well as donor symptoms, and highlight this information when organ offers are made [67]. This protocol suggests that concern for Zika should not summarily exclude donors from transplantation. Rather, the risk of donor derived infection should be balanced with the benefits of the procedure in each potential recipient. In the case of potential living donors with Zika infection, donation should be deferred where possible.

Other guidelines and most published studies suggest donor deferral for 120 days after suspected ZIKV resolution. Based on this data, many experts recommend that SOT/HSCT donors with ZIKV-positive workup, suggestive clinical presentation or travel, and unprotected sexual activity with partner affected by the virus to defer donation for 4 months [54].

### Clinical Manifestations

The most common symptoms in ZIKV infection are maculopapular rash, mild fever, arthralgia, nonpurulent conjunctivitis, myalgia, headache, retro-orbital pain and, less commonly, vomiting, and edema [48••].

Unfortunately, ZIKV infection has been associated with severe congenital complications. Microcephaly is the most devastating feature of congenital infection in infants born to mothers affected by ZIKV during early pregnancy [68••]. In these patients, ocular abnormalities like pigment mottling, chorioretinal, and optic nerve atrophy were found in approximately 35% of cases [69]. Another complication is Guillain–Barre syndrome (GBS), an acute self-limited peripheral neuropathy [70, 71]. Other neurological disorders include acute myelitis and meningoencephalitis [48••, 60]. Some authors suggest that the neurological disorder present in ZIKV-induced GBS can be explained by direct neural injury caused by the virus or by a rapid cellular-mediated response to Zika [71–73].

In the transplantation setting, a report by Nogueira and colleagues describes four SOT/ZIKV+ patients that had concomitant bacterial infections requiring hospitalization. Complications such as graft dysfunction and arterial thrombosis were also observed, but all patients achieved full recovery [59••]. In 2017, a case of ZIKV meningoencephalitis in a heart transplant recipient had a fatal outcome. However, the cause of death was acute cardiac allograft rejection, as a consequence of the abrupt interruption of immunosuppressive drugs due to ZIKV infection [60•].

In oncology and HSCT recipients, Machado and colleagues described a total of four cases of ZIKV. Prolonged viruria and delayed engraftment (HSCT) were observed in ZIKV patients with no further complications [27••]. No ZIKV infection has been reported in the European Society for Blood and Marrow Transplantation registry as of 05/15/2017 [74].

As the symptoms of ZIKV, CHIKV, and DENV are similar, these infections should be considered in the differential of neutropenic fever or rash in post-transplant settings, alongside cytomegalovirus, GVHD, and graft rejection [47, 75].

### Diagnosis

Laboratory confirmation of ZIKV infection can be made through NAT and serology [76]. The definitive diagnosis is established by detection of viral nucleic acid in serum; however, most patients are asymptomatic and exhibit transient viremia (7 to 10 days) making diagnosis elusive. Recent studies have shown that ZIKV RNA persists significantly longer in whole blood than in plasma [77•]. Similarly, Gourinat and colleagues demonstrated that urine samples have higher viral loads for a longer time (> 10 days) when compared to serum. These findings could extend the window during which a definitive diagnosis of ZIKV can be established [78]. Another study compared serum and saliva samples indicating higher sensitivity of the saliva test compared to serum [79].

### Clinical Management

So far, there is no specific therapy for ZIKV infection. Treatment for uncomplicated disease includes rest, hydration, and symptomatic management for fever and arthritis.

The main studies of Zika and chikungunya infections in transplant recipients are shown in Table 1.

**Table 1:** Main studies of Zika and chikungunya infections in transplant recipients

Author	Country	Number of patients	Virus	Population	Comments
Courderc et al. 2012 [37•]	France	4	CHIKV	Corneal grafts	One third of uninfected corneal donors (4 of 12) were infected with CHIKV during the study period.
Dalla-Gasperina et al. 2015 [43]	Italy	1	CHIKV	Kidney transplant	First case of CHIKV infection in an HIV-infected kidney transplant recipient.
Pierrotti et al. 2017 [44]	Brazil	4	CHIKV	Kidney transplant	Described four cases of CHIK among kidney transplant recipients. Immunosuppression may have ameliorated the symptoms
Girão et al. 2017 [45••]	Brazil	13	CHIKV	Kidney and liver transplant	SOT with CHIKV infection appears to have an evolution similar to those seen in the general population.
Machado et al. 2017 [27••]	Brazil	6	ZIKV (4) CHKV(2)	HSCT	Prolonged viruria in ZIKV cases. Delayed engraftment in one patient who acquired ZIKV 25 days before HSCT.
Nogueira et al. 2017 [59••]	Brazil	4	ZIKV	Kidney and liver transplant	Discusses the potential risk of bacterial superinfection in immunocompromised population with ZIKV infection.

## Preventive Measures

Dengue, Zika, and chikungunya prevention is based on vector control and community-based programs to keep the environment free of potential breeding sources (discarded tires, flower vases, uncovered water storage barrels, etc). The mosquitoes breed in standing water [53, 80]. In endemic regions, transplant patients should receive information about arbovirus transmission and *Aedes* habits to avoid exposure. Insect bites should be minimized by protective clothing and application of mosquito repellent (N, N-diethyl-m-toluamide DEET, Picaridin or IR3535). Transplant recipients from non-endemic areas should avoid traveling to endemic areas [29, 31].

As of August 2017, there was one licensed vaccine against dengue and six in development: two in phase III, one in phase II, and three in phase I trials [81]. The licensed Sanofi-Pasteur DENV vaccine is a recombinant, chimeric live-attenuated tetravalent vaccine, to be administered in a 3-dose schedule at 6-month intervals. The vaccine has been licensed for individuals aged 9–45 years living in areas with dengue seroprevalence > 70%. There is a clear benefit to people previously seropositive for DENV, with efficacy at 81.9% CI95 (67.2–90.0) [82]. However, seronegative patients show efficacy of 52.5% CI95 (5.9–76.1%) and increased attributable risk of 5 admissions and 2 severe cases per 1000 vaccinated in 5-year follow-up [81, 82]. The ADE phenomena may explain this observation. In seronegative patients, the vaccine mimics a silent primary infection that provides partially neutralizing antibodies for all serotypes. If the subject later acquires a natural, secondary dengue infection, the disease may exhibit a severe course [81]. According to the WHO, this is the main obstacle to the widespread use of the licensed vaccine, and several questions must be answered before dengue vaccines are introduced broadly [83••].

The other dengue vaccine undergoing phase III clinical trials is the Butantan-DV vaccine, a live-attenuated tetravalent vaccine produced by the NIH and Butantan Institute. The results of a safety and efficacy trial (ClinicalTrials.gov identifier NCT01696422) in a DENV seropositive patient showed a robust expansion (~ 70-fold) of the plasmablast population post-vaccination, generating neutralizing titers for all serotypes by 91 days and amnestic response to DENV3 [84].

Until data becomes available from forthcoming studies, there is no recommendation concerning the use of dengue vaccines in immunocompromised individuals. Similarly, at this time, there is no recommendation for vaccination of travelers or health-care workers [83••].

Vaccines against Zika and chikungunya are still in early development and need to be tested for efficacy. ZIKV vaccination studies have been conducted in animal models, but no commercial vaccine is currently available [29, 85].

## Conclusions

Clinical manifestations of CHIKV, DENV, and ZIKV infections are similar in transplant recipients. Some findings such as thrombocytopenia and arthralgia are more frequent in dengue and chikungunya, respectively, but do not ensure diagnosis. Fever, rash, nausea, vomiting, leucopenia, thrombocytopenia, and transient alterations of liver or renal functions are common findings in this population. Thus, epidemiological risk and laboratory workup is mandatory to diagnose arboviruses in transplant recipients living in or returning from endemic areas.

Serological diagnosis is currently a challenge, as recent data showed cross-reactivity of dengue tests in patients with proven ZIKV. The specificity of current dengue tests should be urgently reassessed. In addition, due to the high frequency of asymptomatic cases, DENV, CHIKV, and ZIKV may represent a threat to transplant recipients, as they can also be transmitted by blood transfusions or organ/tissue transplantation. So far, NAT is preferred in blood bank and pre-transplant donor selection, as well as in symptomatic or suspect cases after transplantation [86, 87]. Further research is needed to define the blood component to be tested (plasma, serum, whole blood) to ensure safety.

The burden of these diseases is unknown in the transplant setting, as only one prospective study has been conducted in symptomatic patients. Among the three discussed arbovirus infections, DENV showed greater morbidity with a protracted course, prolonged viremia and more complications.

No specific treatment is currently available and vaccines are in development. Management of arboviruses is mainly supportive, with attention to warning signs of decompensation.

Reduction of immunosuppression is not recommended as it may trigger rejection or GVHD. Preventive measures include vector control, avoidance of mosquito bites, and hopefully soon, vaccination.

The immunological consequences of DENV, CHIKV, and ZIKV persistence in blood and tissues, as well as its impact on the graft, need further investigation.

The neglected status of these diseases and lack of appropriate research funding poses a significant challenge for better understanding the many knowledge gaps in the field. Arboviruses are not contained by man-made borders. More research is urgently needed to elucidate the morbimortality of arbovirus in transplant cohorts including the variables associated with severe forms of the diseases.
